# Design and Synthesis of Small Molecules as Potent *Staphylococcus aureus* Sortase A Inhibitors

**DOI:** 10.3390/antibiotics9100706

**Published:** 2020-10-16

**Authors:** Min Woo Ha, Sung Wook Yi, Seung-Mann Paek

**Affiliations:** 1 College of Pharmacy, Jeju National University, 102, Jejudaehak-ro, Jeju, Jeju-do 63243, Korea; minuha@jejunu.ac.kr; 2 Interdisciplinary Graduate Program in Advanced Convergence Technology & Science, Jeju National University, 102, Jejudaehak-ro, Jeju, Jeju-do 63243, Korea; 3 Department of Chemistry & Biochemistry, University of California Los Angeles, CA 90095, USA; woogie@chem.ucla.edu; 4 College of Pharmacy and Research Institute of Pharmaceutical Sciences, Gyeongsang National University, Jinju Daero 501, Jinju, Gyeongsangnam-do 52828, Korea

**Keywords:** drug resistant, sortase A, *Staphylococcus aureus*, sortase A inhibitor, small organic molecule, synthetic chemistry

## Abstract

The widespread and uncontrollable emergence of antibiotic-resistant bacteria, especially methicillin-resistant *Staphylococcus aureus*, has promoted a wave of efforts to discover a new generation of antibiotics that prevent or treat bacterial infections neither as bactericides nor bacteriostats. Due to its crucial role in virulence and its nonessentiality in bacterial survival, sortase A has been considered as a great target for new antibiotics. Sortase A inhibitors have emerged as promising alternative antivirulence agents against bacteria. Herein, the structural and preparative aspects of some small synthetic organic compounds that block the pathogenic action of sortase A have been described.

## 1. Introduction

Antibiotics are indispensable to the health of humankind [1,2,3]. Owing to the great contribution of commendable antibiotics, we might expect to extend our life expectancy over the age of 50 without substantial difficulties. Antibiotics deactivate bacterial infections by directly killing the bacteria and/or blocking its growth, ultimately preventing its spread [4]. However, the disconcerted emergence of antibiotic-resistant bacteria [5], which are resistant to several conventional antibiotics including β-lactam, cephalosporin, tetracycline, fluoroquinoline, macrolide, vancomycin, aminoglycoside, and trimethoprim-sulfamethoxazole, has become a serious social problem [6,7,8,9]. The greatest concerns regarding antibiotic-resistant bacteria, especially methicillin resistant *Staphylococcus aureus* (MRSA) for several difficult-to-treat infections in humans such as pneumonia, bacteremia, osteomyelitis, and endocarditis [10,11], resulted in the development of a new generation of antibiotics that target the alternative enzyme components, fighting bacterial infections not as bactericides or bacteriostats [12,13].

Since the elucidation of sortase, one of the membrane-bound cysteine transpeptidase in Gram-positive bacteria, in 1999, it has been regarded as a great target in the development of novel types of antibiotics. Its merits include: (1) its location on the extracellular membrane, (2) its lack of homologs in humans, and (3) its irrelevancy to bacterial growth [14,15]. Among the 1000+ homologs, sortase A in *Staphylococcus aureus* is known as the prototypical subtype [16,17]. To date, several research works have been published on the structure and mode of action of sortase A in *S. aureus* [18]. In terms of new drug design and discovery, a major achievement is associated with the development of sortase A inhibitors as anti-infective agents [19,20]. Sortase A inhibitors could be free from the consideration of their penetration inside the bacterial membrane. Sortase A inhibitors reduce the bacterial virulence caused by adhesion of the surface protein that carries disease-causing components to the host. Since sortase A is not required for the growth of *S. aureus*, drug resistance owing to sortase A inhibitors is less expected [21,22,23,24,25,26,27,28,29,30,31,32,33,34,35].

**Figure 1:**
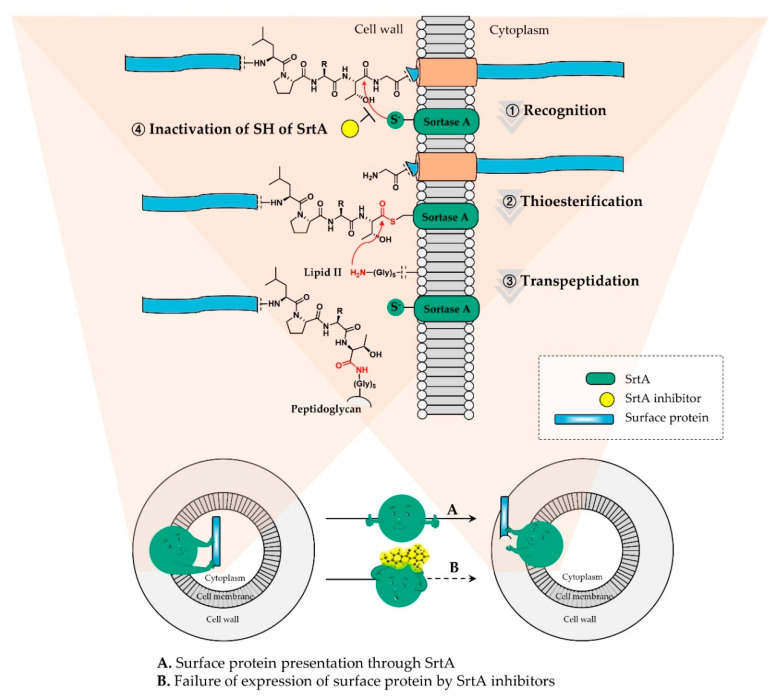
Mode of action of SrtA and its inactivation through its inhibitor; the surface proteins are covalently attached to the cell wall peptidoglycan with the aid of SrtA via the following sequential steps: (1) recognition, (2) thioesterification, and (3) transpeptidation. Further, they are inhibited for display, resulting in failure of adhesion to specific organ tissues, invasion of host cells, or the evasion of host-immune responses (4).

The enzymatic mode of action of sortase A commenced with the recognition step of the C-terminal amino acid sequence LPXTG (X means any amino acids) motif in the target surface protein. Sulfhydryl functionality of Cys184, one of the conserved residues in the catalytic domain, attacks nucleophilically the carbonyl carbon of Thr in the cell wall sorting signal, breaks down the Thr and Gly linkage, and affords the thioester intermediate. This covalent acyl-enzyme adduct undergoes another nucleophilic acyl substitution by the amino group of Gly in lipid II, forming the rigid amide bond; by virtue of these series of steps of sortase A, the surface protein is incorporated into the peptidoglycan of the cell wall. Therefore, the most reliable strategy for inhibition of the enzyme is to inactivate the thiol functional group with a variety of electrophilic molecules covalently or non-covalently. Figure 1 displays the mechanism of sortase A (green) and its inactivation process through a sortase A inhibitor (yellow).

With the attractive factors of sortase A, several strategies have been explored to isolate single ingredients with inhibitory activities against sortase A from natural products and compound libraries [36,37]. The design and synthesis of new molecules inspired by their own leading skeleton have also been discussed. Several synthetic small organic compounds constructively prepared by chemists were selected and described in this review. Further, a brief history of the molecular design and arrangement of a carbon skeleton is presented.

## 2. Results and Discussion

**Figure 2:**
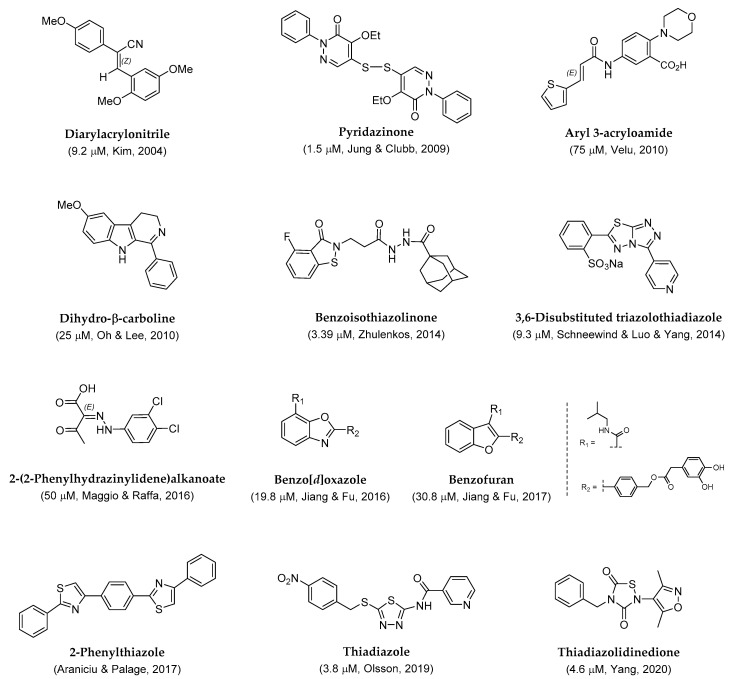
Synthetic small organic molecules as SrtA inhibitors (IC_50_ values are given in parentheses).

To explore the practicality of sortase A inhibitors, high-throughput screening (HTS) has been preliminarily adopted for constructive syntheses. In addition, further structural modifications through structure–activity relationship analysis have been performed to identify potential skeletons and functionalities. Several significant small organic molecules have been designed and synthesized, as shown in Figure 2, with promising IC_50_ values. Herein, the concrete synthetic features and the analytic properties for structural identification are described in the order of diarylacrylonitriles [38], pyridazinones [39], aryl 3-acryloamides [40], dihydro-β-carboline [41], benzisothiazolinones [42], triazolothiadiazoles [43], 2-(2-phenylhydrazinylidene)alkanoates [44], 2-phenyl-benzo[*d*]oxazole-7-carboxamide [45], 2-phenyl-benzofuran-7-carboxamide [46], 2-phenylthiazoles [47], 2, 5-disubstitued thiadiazole [48], and thiadiazolidinedione [49].

### 2.1. Preparation of Diarylacrylonitriles

**Scheme 1:**
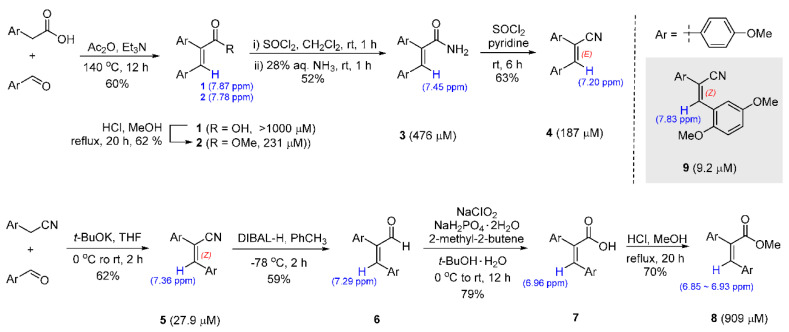
Geometrically selective synthesis of diarylacrylonitriles (IC_50_ values are given in parentheses).

Through random screening with the diverse small molecular library of 1000 compounds, a molecule of *E*-configurated diaryl acrylate was discovered with an IC_50_ of 231 µM by the Kim research group [38]. With this attractive starting lead, a series of structural modifications such as *E*/*Z*-geometric isomerism, the absence of double bond, and substitution of ester for other functionalities was performed, which finally afforded *Z*-diarylacrylonitriles (**5** and **9**) with high inhibitory potencies against *S. aureus*. To prepare the two types of double bond structures, different synthetic strategies were used to obtain the corresponding isomer, as illustrated in Scheme 1 [50].

The molecular geometry of the acrylic acid **1** condensed with *p*-methoxyphenyl acetic acid and *p*-anisaldehyde in acetic anhydride and trimethylamine was assigned the configuration *E*, while acrylonitrile **5**, the adduct of *p*-methoxyphenyl acetonitrile and *p*-anisaldehyde under alkoxide basic condition, was synthesized with the entire *Z*-isomerism. Further chemical replacement via substitution, reduction, or oxidation was investigated and a few diarylacrylonitrile derivatives (**2**–**8**) were prepared. These simple and concise synthetic routes were adopted to obtain the best analog **9** with 2,5-dimethoxybenzaldehyde. The NMR spectroscopic analysis of olefinic hydrogen (colored in blue) is used to distinguish its *E*/*Z* geometry [51,52]; the hydrogen in the *Z*-configurated olefin appears to be slightly more upfield while the *E*-geometric hydrogen appears more downfield to the left. Even in this assignment, the comparison of **1** and **7**, **2** and **8**, and **4** and **5** revealed distinguishable information about the carbon skeletons [53].

### 2.2. Preparation of Pyridazinones

Three types of promising heterocycle ring skeletons, rhodanine, pyridazinone, and pyrazolethione, were introduced by the Jung and Clubb group to the HTS, with over 30,000 organic molecules having potential IC_50_ values of up to 3–5 μM [39]. In particular, a further investigation of pyridazinone derivatives resulted in disulfide **15**, which had an IC_50_ value of 1.5 μM. Its potency was understood via thiol–disulfide exchange reaction with the SH residue of cysteine of sortase A.

**Scheme 2:**
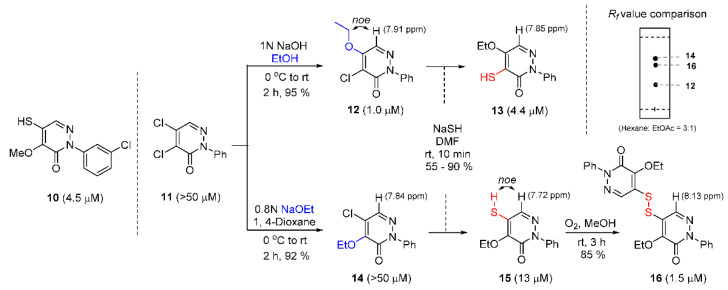
Synthetic methods for pyridazinone derivatives via regioselective substitution (IC_50_ values are given in parentheses).

With a starting standard **10,** the authors intended to investigate the influence of the position of the thiol moiety and proceeded to develop distinct synthetic methods for regioselective substitution with alkoxide to the α or β position of unsaturated ketone. If the relatively mild reaction condition with 1 N of sodium hydroxide in ethanol proceeded with substrate **11**, only β-substituted ethoxide was prepared. However, treatment with ethoxide anion generated in situ 1, 4-dioxane to **11,** predominantly providing the α-substituted counterpart. Moreover, the selective characteristics may depend on the length of the alkoxide carbon chain. Thus, their regioselectivities were determined through the nOe analysis between adjacent hydrogens in a molecule; these structural isomers showed their distinguishable identities on TLC analysis, as shown in Scheme 2. The remaining chlorine atoms of **12** and **14** were fairly quickly replaced with thiol **13** and **15** and thiol molecule **15** was oxidized to disulfide **16** in the presence of oxygen gas [54,55].

### 2.3. Preparation of Aryl 3-Acryloamide

**Scheme 3:**
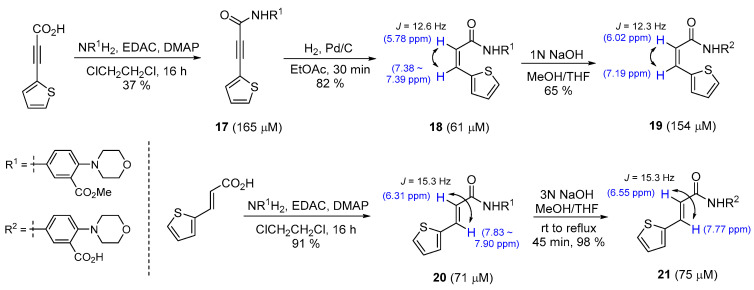
Geometrically selective synthesis of aryl 3-acryloamides (IC_50_ values are given in parentheses).

A series of synthetic molecules, presented as aryl 3-acryloamides, were prepared and their enzyme inhibiting actions were determined by the Velu research group [40]. The authors had performed systemic SAR studies that revealed the promising organic materials formed with the morpholinobenzoate group. To examine the effect of the geometry of the double bond on their activity, Z-configurated olefin molecules, **18** and **19**, and E-configurated molecules, **20** and **21**, were prepared using the optimized reaction methods presented in Scheme 3. As a result, the E stereochemistry was recognized to be more important than the Z configuration or rigid triple bond. Partial reduction with Lindlar catalyst, which is a well-established method for the synthesis of cis-olefin, did not provide the molecule **18,** presumably due to the poisonous nature of the sulfur atom on the thiophene for palladium chemistry. However, the use of H_2_ gas in the presence of Pd/C afforded the desired cis-alkene **18**. For the synthesis of trans-isomer, the authors utilized the already-established cis-compound as the reactant [56,57].

The stereochemistry of E/Z isomers can be elucidated through ^1^H NMR analysis. On the ^1^H NMR spectrum, the olefin hydrogens in **18** and **19** have shown different chemical shifts and coupling constants compared to those of **20** and **21**. As expected, the apparent coupling constants were smaller in the cis-isomers than the trans-isomers. Further, it has been found that the olefinic hydrogens of the trans-diastereoisomer are located downfield than those of the cis-diastereoisomer to a certain degree.

### 2.4. Preparation of Dihydro-β-Carboline

**Scheme 4:**
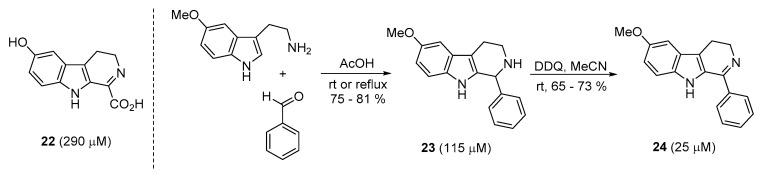
Synthetic methods for tetrahydro-β-carboline and dihydro-β-carboline (IC_50_ values are given in parentheses).

A new heterocyclic skeleton, β-carboline, inspired by the structures of indole-containing natural products, was synthesized by the Oh and Lee group [41]. With 6-hydroxydihydro-β-carboline (**22**) as a standard and through a simple and fast synthetic route [58,59,60,61], the best inhibitor candidate **24** with an IC_50_ value of 25 μM was observed. Briefly, condensation between 5-methoxy tryptamine and benzaldehyde followed by ring closure was very smoothly performed according to the Pictet–Spengler reaction, affording the tetrahydro-β-carboline **23**. Further, oxidation with the aid of the DDQ reagent resulted in the generation of dihydro-β-carboline **24** with good yield. As depicted in Scheme 4, replacement of carboxylic acid with a phenyl ring induced a marked increase in the inhibitory activity of sortase A.

### 2.5. Preparation of Benzoisothiazolinones

**Scheme 5:**
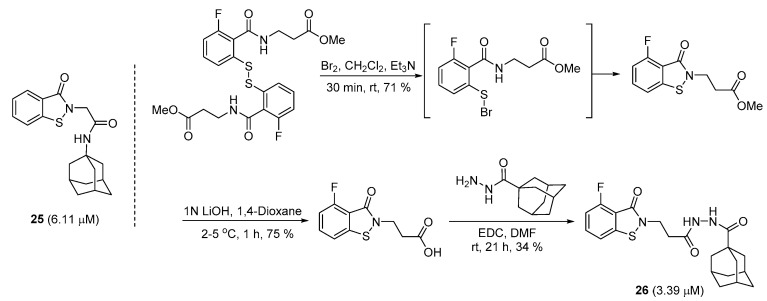
Synthetic routes for benzisothiazolinone-based inhibitors (IC_50_ values are given in parentheses).

From a small molecule library screening that included over 50,000 drug-like compounds, a new class of sortase A inhibitors, with **25** as a representative, was identified by the Zhulenkovs group [16]. The skeleton was comprised of benzo[d]isothiazol-3-(2H)-one heterocycle, which might form a covalent bond with the sortase A enzyme, the adamantyl moiety that offers the hydrophobic property, and an adequate linker. Owing to the results of the SAR analysis, the authors synthesized different combinations of the two ring moieties with 4–6 carboned spacers, which ultimately resulted in an IC_50_ value of ~3 μM (**26**). The core benzoisothiazolinone structure was prepared with the intermediate, 2-carbamoylbenzenesulfenyl bromide, via the treatment with bromine to disulfide. Additionally, the attachment of the adamantyl functionality was completed through evident EDC coupling (Scheme 5) [62,63,64].

### 2.6. Preparation of Triazolothiadiazoles

**Scheme 6:**
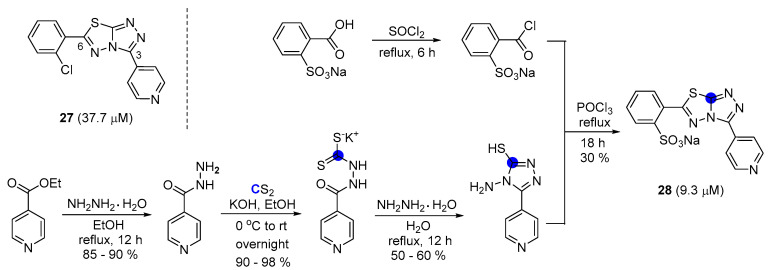
Optimized synthetic scheme of the 3, 6-disubstituted triazolothiadiazole scaffold (IC_50_ values are given in parentheses).

After the acquisition of the hit compound **27** with a 3,6-disubstituted triazole structure, a synthetic optimization was performed by Schneewind, Luo, and Yang group [43], with few modifications, to discover the improved activity. The ethyl isonicotinate nucleophilic substitution with hydrazine was observed to occur as planned. Thereafter, isonicotinohydrazide was condensed with carbon disulfide in the presence of potassium hydroxide, producing potassium dithiocarbazate, and a triazole ring was formed upon treatment with hydrazine hydrate. Ultimately, the thiadiazole structure in **28** was achieved with 4-amino-5-aryl-3-mercapto-1,2,4-triazole and the corresponding benzoyl chloride in the presence of phosphorus oxychloride (Scheme 6) [65].

### 2.7. Preparation of 2-(2-Phenylhydrazinylidene)Alkanoates

**Scheme 7:**
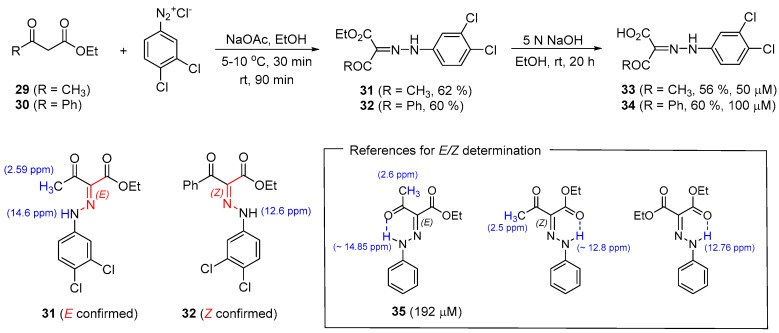
Synthetic methods for 2-(2-phenylhydrazinylidene)alkanoates (IC_50_ values of **33** and **34** are given in parentheses).

The Maggio and Raffa research group has studied the 1,3-dicarbonyl core skeleton with an aryl hydrazinylidene at the α-position of β-keto ester as a novel class of sortase A inhibitors [44]. As shown in Scheme 7, the diazonium salt prepared from the substituted aniline was reacted with β-keto ester **29** or **30** [66,67]. Thereafter, basic hydrolysis was carried out, affording the acids **33** and **34**. During the synthesis of a series of analogs of the screened molecule, **35**, with an IC_50_ value of 192 μM, an issue ensued regarding the assignment of the geometry of the C=N double bond for **31** and **32** [68,69,70,71]. Through a comparison of the ^1^H NMR spectroscopic information for the reference molecules, the obtained materials, **31** and **32**, were confirmed to be E and Z, respectively [72,73,74]. For the E-configurated molecule, **31**, the hydrogen of NH and the oxygen of the acetyl moiety form intramolecular hydrogen bonding, depicting an apparent downfield shift of NH and methyl signals compared to the Z-geometry [75,76].

### 2.8. Preparation of Benzo[d]Oxazoles and Benzofuran

**Scheme 8:**
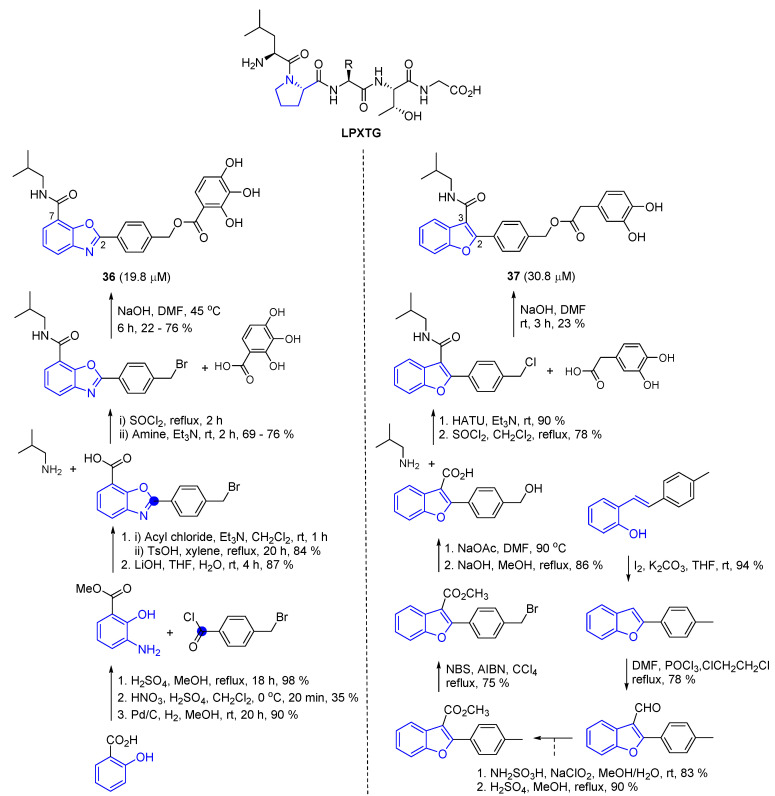
Construction of the benzo[d]oxazole and benzofuran skeleton molecules (IC_50_ values are given in parentheses).

During the search for a new frame of sortase A inhibitors, the Jiang and Fu research group developed L-shaped kinked molecules that mimic the sortase A substrate, LPXTG, sequentially designing 2-phenyl-benzo[d]oxazole-7-carboxamide and 2-phenyl-benzofuran-3-carboxamide [45,46]. Sortase A is known to recognize the LPXTG sorting signal (X means any amino acids) which is arranged in an L-shape mode by the L (Leu) and P (Pro) residues. The authors introduced an isobutyl amide functionality at the C-7 position of benzo[d]oxazoles and the C-3 position of benzofuran. Further, the core bicyclic rings were regarded as the displacement of pyrrolidine, and a right-side assembly of carbons and oxygens was expected to produce some spatial effect, mimicking the X amino acids in the substrate. The constructive methods of the objects are described in Scheme 8 [77].

In order to prepare the molecule **36**, the authors used salicylic acid as a starting material. Following the protection of carboxylic acid to methyl ester of salicylic acid, a nitration reaction was carried out to introduce a nitryl group to an aryl ring [78]. Transformation of the nitryl functionality to amine under a hydrogen atmosphere in the presence of Pd/C enabled the 2-hydroxyl aniline to react with 4-methyl benzoyl bromide via intramolecular cyclization, resulting in benzoxazole. The deprotected free carboxylic acid derived via hydrolysis was then converted to acyl chloride. Thereafter, nucleophilic substitution with isobutyl amine was performed, followed by a final connection of the ester component via an undoubtful S_N_2 reaction [79].

To synthesize 2,3-substituted-benzofuran **37**, (E)-2-(4-methylstyryl)phenol prepared via Wittig olefination was treated with iodine under basic conditions. Ring closure by iodination was then followed by a Vilsmeier reaction, resulting in a formyl moiety at the C-3 position. Following stepwise reactions, oxidation, esterification, bromination, hydrolysis, amide coupling, and substitution were performed.

### 2.9. Preparation of Phenylthiazoles

**Scheme 9:**
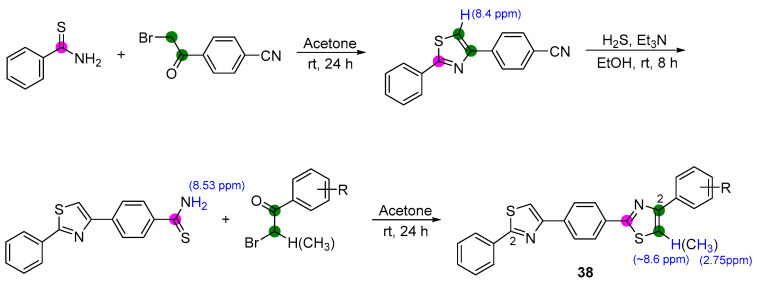
Synthesis of 2-phenylthiazole derivatives within a pentacyclic system.

A study on the structural investigation of sortase A inhibitors through Bemis–Murcko scaffold analysis revealed that the scaffolds preferred for sortase A inhibitors contain at least two ring systems bonded directly or merged in a condensed cycle (Scheme 9). The Araniciu and Palage group designed a new framework of five aromatic rings linked directly and inspired by Bemis–Murcko clustering, ultimately synthesizing a series of 2-phenyl-thiazole derivatives **38** [47]. Briefly, thiobenzamide and 4-(2-bromoacetyl)benzonitrile were intramolecularly cyclized in one pot, affording the key intermediate, 4-(2-phenylthiazol-4-yl)benzonitrile. Treatment with hydrogen sulfide gas under triethylamine condition transformed nitrile into thioamide via a further thiazole ring formation reaction. Structures of the molecules were confirmed based on the distinct hydrogen signals on the ^1^H NMR spectra and sharp signals between 3111 and 3103 cm^−^^1^ on the IR spectra [80]. Although the authors have not demonstrated the IC_50_ values of these sorts of new inhibitors, the antimicrobial activity evaluation showed that the potential scaffold has reduced activity against bacterial cell viability.

### 2.10. Preparation of Thiadiazole

**Scheme 10:**
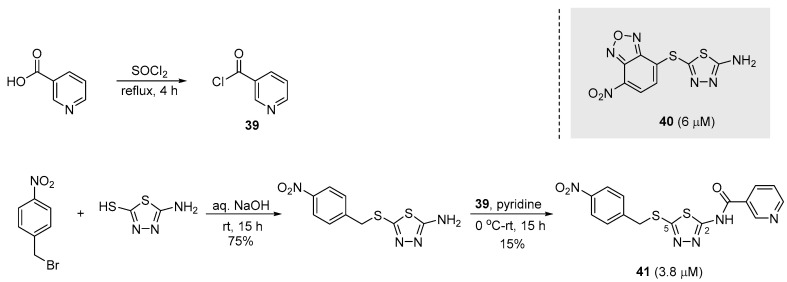
Synthetic process for 2, 5-substituted thiadiazole (IC_50_ values are given in parentheses).

The Olsson group identified a new class of inhibitors against S. aureus sortase A [48]. Through small-molecule libraries screening and PAINS (pan-assay interference compounds) study, top hit structures emerged. The hit compound **40** was selected as the starting point for structural hit-to-lead optimization. A series of modifications of **40** commenced with the removal of the oxadiazole unit which has been shown to cause cytotoxicity. Synthetic modifications included elongation of the sulfide substituent and addition of various substituted benzyl derivatives to validate some electronic effects. After all, N-(5-((4-nitrobenzyl)thio)-1,3,4-thiadiazol-2-yl)nicotinamide **41** (IC_50_ = 3.8 μM) was identified as a potent inhibitor of sortase A. As illustrated in Scheme 10, 5-amino-1,3,4-thiadiazole-2-thiol was reacted with the appropriate bromide under basic conditions, affording the desired products in good yields. The primary amine was acylated, which was achieved upon treatment with nicotinoyl chloride **39** that was induced from nicotinic acid in situ [81,82,83].

### 2.11. Preparation of Thiadiazolidinedione

**Scheme 11:**
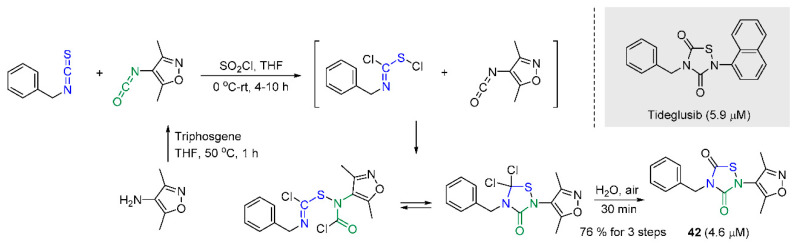
Construction of the thiadiazolidinedione scaffold through chloro-intermediates (IC_50_ values are given in parentheses).

In an attempt to discover innovative scaffolds for sortase A inhibitors, the S. Yang and C.-G Yang research groups employed FRET-based HTS on a library consisting of over 2400 clinical drugs and candidates [49]. Tideglusib (TD), a drug candidate for myotonic dystrophy [84], was identified as a hit structure with an IC_50_ value of 5.9 μM. The authors prepared 40 sorts of TD analogs which were constructed with a thiadiazolidinedione skeleton and analyzed their structure–activity relationships for sortase A inhibition. Several molecules were explored, changing two N-substitutions with a variety of functionalities [85], and it was observed that the most promising compound, **42**, bears 3,5-dimethylisoxazole substituent, retains inhibitory activities compared to that of TD, shows better solubility, and minimally inhibits the growth of the S. aureus. When it comes to the preparation of TD analogs, a concise synthetic route illustrated in Scheme 11 was adopted. The reaction system with an isothiocyanate, an isocyanate, and same equivalent molar amount of sulfuryl chloride (SO_2_Cl_2_) even generated the unstable S-chloroisothiocarbamoyl chloride, and via the assembly with chloro-intermediates, the desired scaffold was established with good conversion efficiency.

## 3. Conclusions

Novel approaches for the development of antibiotics includes targeting virulence via toxin production and virulence factor secretion, impeding bacterial adhesion to host cells and biofilm formation, interrupting or inhibiting bacterial communication, and downregulating virulence. As part of the struggle against drug resistance, the development of sortase A inhibitor has emerged as an alternative to conventional antibiotics, with a mechanism that involves blocking the pathogenic processes of bacterial infection. The ideal inhibitors for sortase A should have minimal impacts on bacterial viability, which can be validated by their minimal inhibitory concentration (MIC) test. A number of influential advances in the development of sortase A inhibitors were comprehensively studied and reviewed by a variety research groups, but the interest and focus on their structural and preparative aspects are insufficient so far. It is of great interest to the medicinal chemists and associates how the core scaffolds are established and the tangent molecular groups are chemically installed. We the authors focus on the synthetic area and highlight significant small organic molecules with high inhibitory potencies against S. aureus, eliciting the greatest IC_50_ values up to 1.5 μM.

The organic molecular group, aryl(β-amino)ethyl ketones, mainly abbreviated as AAEK and one of the promising S. aureus sortase A inhibitors, is known to their inhibitory mechanism [86]. In virtue of the kinetic analysis and mass spectrometry, it is suggested that the initial deprotonation of α-position of AAEK by an appropriate base of sortase A facilitates the generation of an enolate which is stabilized by the guanidinium residue in the enzyme and then β-elimination, which means that the cleavage of the C-N bond is undergone, releasing the separate moiety dimethylamine. Therefore, AAEK is now considered as an α,β-unsaturated ketone at which 1,4-Michael type addition can be easily performed by the nucleophilic attack of the thiol group of cysteine in sortase A [87,88,89,90]. Through the irreversible covalent bond formation between sortase A and its inhibitor, the enzymatic action was attenuated. In a similar context, the α,β-unsaturated carbonyl system of sortase A inhibitors is regarded as a Michael donor. Moreover, there are some sulfur containing sortase A inhibitors which inhibit sortase A by forming a disulfide bond with the cysteine residue, and it seems that most of the potent chemicals for sortase A bear sulfur (S) atoms in the molecules.

In summary, high-throughput screening and in silico virtual screening techniques from the small-molecule libraries have been widely used to discover novel types of scaffold sortase A inhibitors. As discussed above, we can determine the tendency that many compounds have α,β-unsaturated carbonyl core or sulfur-associated functionalities. In the text, 12 sorts of skeletons, diarylacrylonitriles, pyridazinones, aryl 3-acryloamide, dihydro-β-carboline, benzisothiazolinones, triazolothiadiazoles, 2-(2-phenylhydrazinylidene)alkanoates, 2-phenyl-benzo[d]oxazole-7-carboxamide, 2-phenyl-benzofuran-7-carboxamide, 2-phenylthiazoles, 2, 5-disubstitued thiadiazole, and thiadiazolidinedione, are demonstrated from a synthetic point of view. These cumulative achievements regarding sortase A inhibitors have provided the molecular basis for designing and synthesizing sortase A inhibitors. Their systematic development is thus expected to result in a novel class of antibiotics that would serve as antivirulence agents in the near future.
